# Supplementary material for: Viral capsid delivery of cGAMP enhances STING-dependent antitumor immune response
Source: bioRxiv. 2026 Jul 1:2026.06.26.734859. Preprint. [Version 1] doi: 10.64898/2026.06.26.734859 (PMC13345200; doi:10.64898/2026.06.26.734859)
Supplement: Supplement 1 [file NIHPP2026.06.26.734859v1-supplement-1.pdf]

bioRxiv preprint doi: https://doi.org/10.64898/2026.06.26.734859; this version posted July 1, 2026. The copyright holder for this preprint (which was not certified by peer review) is the author/funder, who has granted bioRxiv a license to display the preprint in perpetuity. It is made available under a CC-BY 4.0 International license.

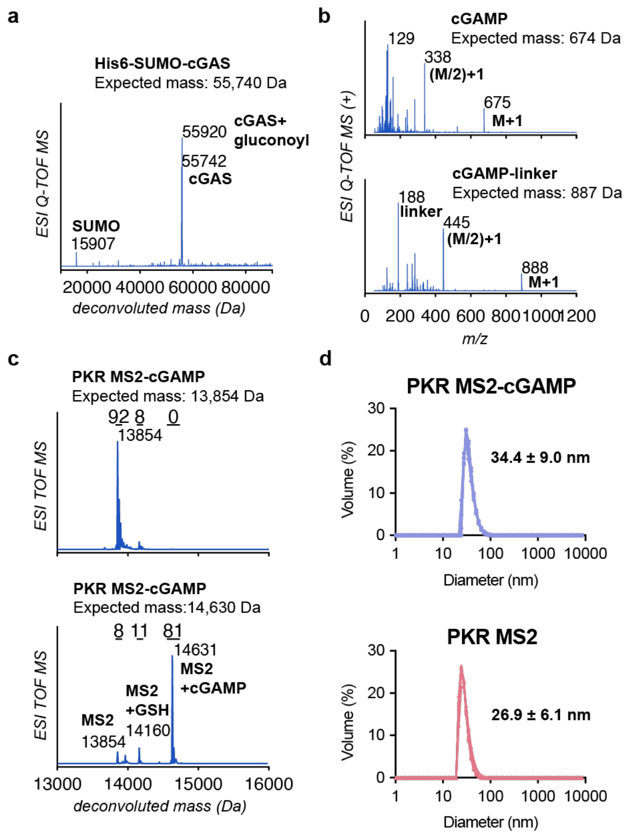

Figure S1. Construction and assembly of PKR MS2-cGAMP and components. **(a)** LC/MS spectrum of human truncated cGAS protein, expressed with a hexahistidine-SUMO tag for ease of purification. **(b)** LC/MS spectra of 2',3'-cGAMP produced enzymatically as well as cGAMP-disulfide conjugate **2** (from **Scheme S1**) produced synthetically. **(c)** LC/MS spectra of PKR MS2 before and after conjugation to cGAMP. **(d)** Dynamic light scattering (DLS) spectra of PKR MS2 before and after attachment of cGAMP.

bioRxiv preprint doi: https://doi.org/10.64898/2026.06.26.734859; this version posted July 1, 2026. The copyright holder for this preprint (which was not certified by peer review) is the author/funder, who has granted bioRxiv a license to display the preprint in perpetuity. It is made available under a CC-BY 4.0 International license.

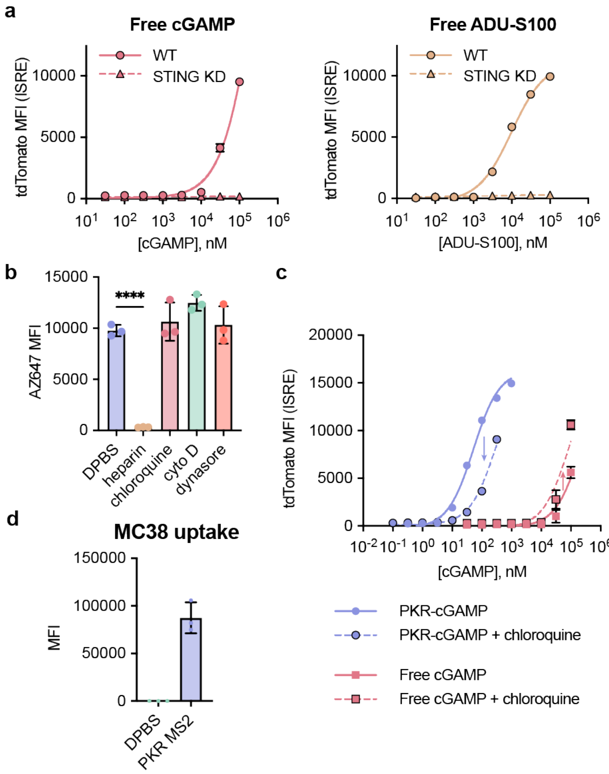

Figure S2. *In vitro* uptake and STING activation effects. **(a)** Type I IFN response in THP-1 reporter cells with wild-type STING (WT) was compared to that in STING knockdown (STING KD) reporter cells. **(b)** THP-1 STING reporter cells were treated with 200 nM PKR MS2 conjugated to a fluorescent AZFluor 647 maleimide dye in the presence or absence of various uptake and endocytosis inhibitors. Cells were analyzed for uptake by flow cytometry. **(c)** THP-1 STING reporter cells were treated with PKR MS2-cGAMP or free cGAMP in the presence or absence of 50 µM of the endosomal acidification agent chloroquine. Cells were analyzed for STING activation by flow cytometry. **(d)** MC38 cells were treated with 200 nM PKR MS2-dye conjugate or control and analyzed by flow cytometry for uptake.

bioRxiv preprint doi: https://doi.org/10.64898/2026.06.26.734859; this version posted July 1, 2026. The copyright holder for this preprint (which was not certified by peer review) is the author/funder, who has granted bioRxiv a license to display the preprint in perpetuity. It is made available under a CC-BY 4.0 International license.

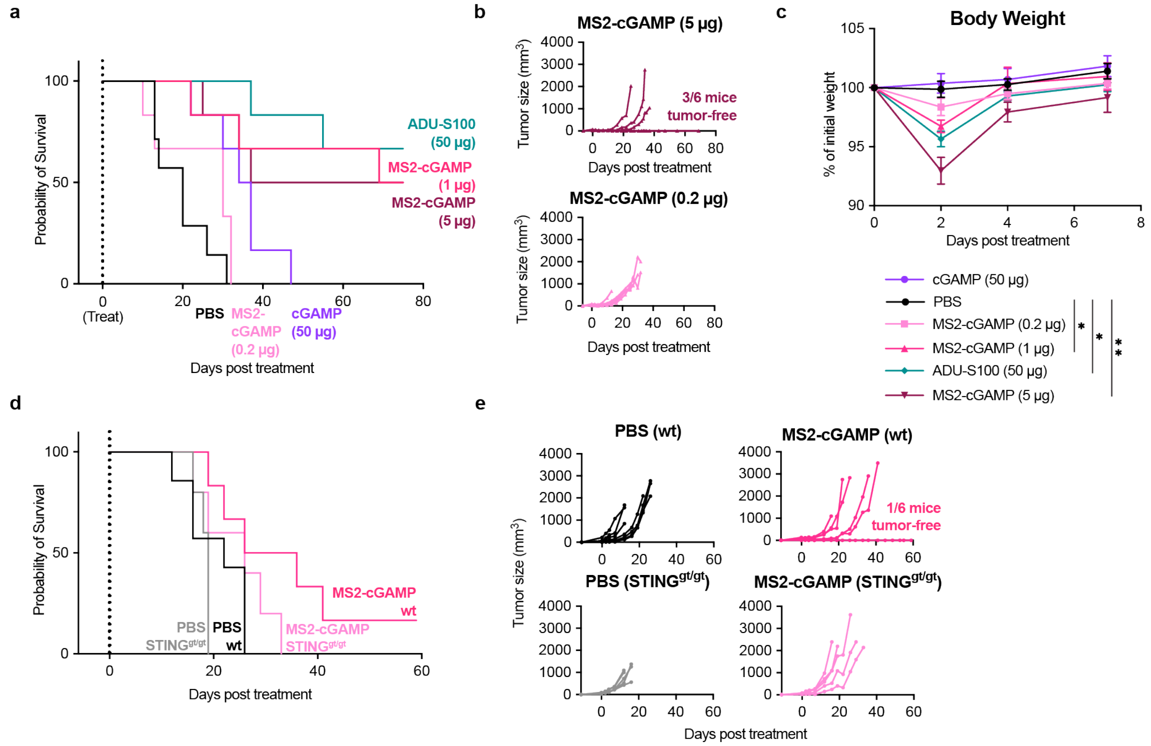

Figure S3. *In vivo* antitumor efficacy of PKR MS2-cGAMP. **(a)** Kaplan-Meier survival curves for mice treated with a single injection of 0.2 µg and 5 µg PKR MS2-cGAMP. Curves for mice cohorts shown in **Figure 4c** are also shown for comparison. **(b)** Individual tumor volumes over time for 0.2 µg and 5 µg PKR MS2-cGAMP dosage cohorts. **(c)** Relative body weight of all mice treated with PKR MS2-cGAMP, free cGAMP, free ADU-S100, or PBS control. Body weights were compared by ANOVA, with the statistical analysis shown representing significant body weight changes in treatment arms versus PBS control mice on day 2 after treatment. Mice in all treatment arms recovered their body weight within 7 days. **(d)** Kaplan-Meier survival curves for wt C57BL/6J and STING$^{gt/gt}$ mice treated with PKR MS2-cGAMP or PBS control. **(e)** Individual tumor volumes over time for wt C57BL/6J and STING$^{gt/gt}$ mice treated with PKR MS2-cGAMP or PBS control.

bioRxiv preprint doi: https://doi.org/10.64898/2026.06.26.734859; this version posted July 1, 2026. The copyright holder for this preprint (which was not certified by peer review) is the author/funder, who has granted bioRxiv a license to display the preprint in perpetuity. It is made available under a CC-BY 4.0 International license.

TABLES:

Table S1. Tumor volumes in mice from the *in vivo* tumor response study represented in **Figure 4b**. All values are given in mm^3. Each column represents the tumor volumes of one mouse in each sample. Mice that were sacrificed or died during the study are marked with an asterisk (*), while mice with no measurable tumor volume are indicated with a zero (0). The study was concluded 69 days after STING agonist or control treatment.

Day	PBS						
0	54.0514786	54.0665582	16.3122706	34.3920452	7.48989199	102.348439	38.7452172
2	52.5699977	80.8063969	26.3459405	51.0926261	16.589114	104.449002	67.7283394
4	60.9410499	81.8864293	18.7628312	57.7584794	18.4701248	106.994268	72.0674862
7	134.239271	179.548125	44.2356247	158.522931	36.0445921	196.66622	137.273429
10	192.092094	240.324377	133.242798	315.594298	146.953688	319.295116	282.266185
13	383.126649	385.222952	274.017502	484.753697	316.069982	627.626574	441.857561
16	1058.2113	1062.49436	595.014021	958.968896	215.337117	990.067165	1099.01115
19	2283.67238	1942.13559	1214.42334	1898.93861	392.063742	*	*
22	*	*	*	*	*		
Day	PKR MS2-cGAMP (1 µg cGAMP)						
0	73.6322297	41.2824124	45.3864027	56.837311	22.101185	31.1127126	
2	71.0906844	33.004366	133.961323	63.2464805	31.0055089	22.7637605	
4	40.7561957	18.1521224	111.879922	23.3635596	19.0383641	10.3474658	
7	35.9225412	17.4061983	170.714516	49.0542807	13.6887674	16.9603278	
10	14.4802917	17.3431622	250.551313	66.1670375	10.8745842	7.99184519	
13	9.04751667	6.77676407	430.779848	50.0620329	8.24887983	3.36477663	
16	0	0	615.316611	85.4224175	4.16562096	6.44221273	
19	0	0	1372.12975	214.581263	0	8.85234051	
22	0	0	2658.02037	348.256855	0	10.412813	
25	0	0	*	396.928218	0	15.2585773	
27	0	0		554.695689	0	14.1494569	
30	0	0		808.48914	0	22.5171611	
32	0	0		1431.42378	0	34.8132448	
33	0	0		1713.03776	0	61.0117415	
34	0	0		2537.56283	0	100.878882	
35	0	0		*	0	45.0227926	
37	0	0			0	56.2079024	
43	0	0			0	154.386879	
47	0	0			0	259.665911	
55	0	0			0	1031.37525	
69	0	0			0	2000	
Day	Free cGAMP (50 µg)						

bioRxiv preprint doi: https://doi.org/10.64898/2026.06.26.734859; this version posted July 1, 2026. The copyright holder for this preprint (which was not certified by peer review) is the author/funder, who has granted bioRxiv a license to display the preprint in perpetuity. It is made available under a CC-BY 4.0 International license.

0	62.2423269	42.8009326	61.7776864	54.1679647	25.8244152	28.3569264	
2	66.8365617	29.1437696	57.0599054	38.6877082	19.4034527	48.0887043	
4	41.2835999	11.1450544	29.6641745	37.8296356	18.4805319	42.9275284	
7	49.8600913	10.8999568	52.4029734	41.36148	22.7821597	79.3394899	
10	64.9064163	8.98219039	64.6773267	61.9103967	26.8467942	109.613429	
13	86.643696	12.8519807	102.177276	115.279046	83.7220829	167.63507	
16	168.264197	10.448156	207.05546	195.653435	161.012961	408.442233	
19	367.814326	18.6103299	428.532148	278.380452	317.958465	570.963931	
22	546.755794	33.309754	647.117224	379.306409	493.547523	*	
25	632.045025	34.2623289	969.21124	257.025607	853.224678		
27	463.957513	29.568335	1210.93794	230.146221	1148.7698		
30	669.934329	65.9936776	2400.06829	278.86762	848.860241		
32	893.146283	178.716209	*	564.092291	1006.91588		
33	1258.80515	193.851095		596.671436	1570.62992		
34	1779.52332	245.312079		907.008984	1312.09305		
35	*	225.552806		871.080005	1653.20242		
37		353.878933		935.618137	2147.22024		
43		1449.58353		*	*		
47		2998.12505					
55		*					
Day	**ADU-S100 (50 µg)**						
0	57.3529155	41.8369082	66.700737	25.5413996	44.7218532	29.6368112	
2	49.0513747	16.9261724	54.4067927	13.7754204	21.0636766	22.4607067	
4	38.275858	24.2863333	19.2330124	25.5851557	16.1076932	15.1948721	
7	43.7131674	21.4900687	18.2220416	16.9658303	21.9432341	20.2526792	
10	62.4915322	13.9626201	15.7675614	15.1281185	7.6785551	17.5961359	
13	69.2483346	0	9.23320783	12.070355	10.088075	11.9102888	
16	115.750836	0	5.20991254	9.86616126	9.51836339	4.82792733	
19	183.659014	0	13.9571537	8.24219871	6.74647807	3.14391063	
22	373.754107	0	31.07578	11.3352888	4.49993354	2.06793033	
25	391.522367	0	35.4336214	0	0	0	
27	308.277199	0	60.6463539	0	0	0	
30	339.115787	0	138.418058	0	0	0	
32	652.6298	0	214.564998	0	0	0	
33	810.453815	0	258.913228	0	0	0	
34	1234.03395	0	210.443266	0	0	0	
35	1262.80391	0	142.490881	0	0	0	
37	1448.6075	0	210.589584	0	0	0	
43	*	0	1125.3067	0	0	0	
47		0	272.240215	0	0	0	
55		0	1616.40892	0	0	0	
69		0	*	0	0	0	
Day	**PKR MS2-cGAMP (5 µg cGAMP)**						

bioRxiv preprint doi: https://doi.org/10.64898/2026.06.26.734859; this version posted July 1, 2026. The copyright holder for this preprint (which was not certified by peer review) is the author/funder, who has granted bioRxiv a license to display the preprint in perpetuity. It is made available under a CC-BY 4.0 International license.

0	41.6242303	68.914033	29.924047	45.2144717	25.2072912	57.1914533	
2	45.3826265	35.2180076	32.9980096	61.7274702	26.7094186	26.780227	
4	28.7458555	26.7094228	28.2445956	33.5091153	17.9095914	9.21329432	
7	41.2949285	24.7440006	35.3860781	26.3879792	31.4778185	33.5105991	
10	33.350978	59.3968147	35.6760644	23.3602782	25.9968363	34.5169424	
13	24.0748099	103.617278	30.0948868	4.90728292	3.96113993	17.6416596	
16	18.0802977	268.966479	45.0449487	0	2.29679745	8.54051231	
19	44.0222477	579.530099	168.596096	0	0	0	
22	62.8387017	704.522208	177.34102	2.56856615	0	0	
25	89.9645029	2030.61997	365.631848	0	0	0	
27	180.006624	*	401.109569	0	0	0	
30	295.872264		749.922357	0	0	0	
32	443.506753		1117.90872	0	0	0	
33	553.223722		1429.55269	0	0	0	
34	815.176105		2762.86958	0	0	0	
35	832.725503		*	0	0	0	
37	1048.02372			0	0	0	
43	*			0	0	0	
47				0	0	0	
55				0	0	0	
69				0	0	0	
Day	**PKR MS2-cGAMP (0.2 µg cGAMP)**						
0	42.2225361	31.7740943	16.1766627	46.3015491	103.886971	39.0693908	
2	69.9221449	49.1252508	14.5635858	41.7331556	85.0620318	45.652206	
4	35.3298797	49.3230225	11.0871077	20.6171343	76.6463491	49.309433	
7	49.5222994	86.7529888	23.2355648	38.9884555	89.187687	147.335401	
10	48.3095059	112.05375	30.1016727	43.0452983	131.192514	372.053741	
13	81.267278	198.01292	65.1535948	98.6896398	230.249403	678.059996	
16	231.49543	294.366996	220.221247	137.033651	382.784432	*	
19	419.582177	491.685489	*	322.60743	581.551027		
22	653.337	739.773827		509.028601	789.178655		
25	722.290671	783.844165		921.49081	1010.30469		
27	947.457365	1313.30511		924.870005	1143.27743		
30	*	823.180062		1415.00299	2221.28018		
32		1533.24364		*	2010.18265		
33		*			*		

bioRxiv preprint doi: https://doi.org/10.64898/2026.06.26.734859; this version posted July 1, 2026. The copyright holder for this preprint (which was not certified by peer review) is the author/funder, who has granted bioRxiv a license to display the preprint in perpetuity. It is made available under a CC-BY 4.0 International license.

Table S2. Tumor volumes in mice from the *in vivo* tumor response study represented in **Figure 4e**. All values are given in mm^3. Each column represents the tumor volumes of one mouse in each sample. Mice that were sacrificed or died during the study are marked with an asterisk (*), while mice with no measurable tumor volume are indicated with a zero (0). The study was concluded 59 days after STING agonist or control treatment.

Day	**PBS – STING WT mice**						
0	10.5623157	7.2763815	75.7625856	20.0544195	41.7534942	214.652774	74.2871518
2	28.2256612	24.641082	189.401951	23.1873058	81.781442	403.045306	162.527871
4	19.6768943	41.0051056	334.095863	19.5595433	112.040211	558.626523	226.828714
7	24.6490978	67.7962826	414.994336	54.4096202	150.710041	1067.38715	269.74337
12	148.071636	228.791141	1683.64885	123.517412	361.734288	1560.77209	851.034911
16	325.999206	484.323332	*	275.277669	670.321366	*	*
19	665.780957	765.440561		660.056159	1277.79921		
22	1342.94715	1678.4412		1438.10013	2096.0586		
26	2656.62328	2777.87968		2083.73775	*		
29	*	*		*			
Day	**PKR MS2-cGAMP (2 µg) – STING WT mice**						
0	40.8176326	140.4307	63.2619329	27.2285605	12.6868161		
2	51.0185976	120.965526	73.8720893	22.5182481	22.8280689		
4	68.1537194	148.023544	105.724699	19.665038	9.14206918		
7	166.893905	149.499114	65.8069078	5.93984274	7.31020964		
12	401.174062	267.564772	78.6283778	17.1148077	1.49527244		
16	1099.98338	511.521153	97.5735481	85.1725572	0		
19	*	557.802222	111.778501	111.556896	0		
22		2754.87832	333.299628	308.112194	0		
26		*	305.940922	518.447179	0		
29			616.60313	1030.01174	0		
33			1274.28896	1968.92383	0		
36			1364.10275	2907.97493	0		
41			3500.15493	*	0		
45			*		0		
49					0		
53					0		
55					0		
59					0		
Day	**PBS – STING$^{gt/gt}$ mice**						
0	29.9768258	56.0446935	27.1403655	45.45761	96.5662069		
2	81.6102519	123.91535	75.4917049	89.3109181	156.24945		
4	118.480392	227.297496	122.601439	114.843345	250.879212		
7	223.93459	350.614876	227.2191	250.068783	360.25177		
12	437.115069	1012.97789	724.54677	526.020302	1104.07449		
16	561.101426	*	1259.51752	1386.19644	*		

bioRxiv preprint doi: https://doi.org/10.64898/2026.06.26.734859; this version posted July 1, 2026. The copyright holder for this preprint (which was not certified by peer review) is the author/funder, who has granted bioRxiv a license to display the preprint in perpetuity. It is made available under a CC-BY 4.0 International license.

19	*		*	*			
	**PKR MS2-cGAMP (2 µg) – STING$^{gt/gt}$ mice**						
0	34.7887655	91.305476	14.4942707	38.1326516	77.9818506		
2	80.4151796	147.57918	46.3261431	29.2910789	168.198514		
4	152.519071	204.410918	77.3570381	35.7949606	211.889668		
7	272.760746	271.684933	106.903108	22.6298878	217.742782		
12	720.530659	974.818003	406.303544	158.379555	599.588201		
16	1101.26263	2396.43153	531.960116	240.85363	1080.5039		
19	2191.9424	*	1084.47352	408.515845	1755.665		
22	*		921.758817	327.829152	1802.4092		
26			1912.35986	1052.78442	3623.40168		
29			2395.85191	1596.44215	*		
33			*	2134.94474			
36				*			

bioRxiv preprint doi: https://doi.org/10.64898/2026.06.26.734859; this version posted July 1, 2026. The copyright holder for this preprint (which was not certified by peer review) is the author/funder, who has granted bioRxiv a license to display the preprint in perpetuity. It is made available under a CC-BY 4.0 International license.

MATERIALS AND METHODS:

All reagents were obtained from commercial sources and used without further purification unless otherwise indicated. Dulbecco's phosphate-buffered saline (DPBS) and all mammalian cell growth media was purchased from Gibco. All other aqueous buffers and media were prepared using milli-Q H_2O purified to a resistivity of 18.2 MΩ at 25 °C obtained from a MilliporeSigma Milli-Q EQ7000 purification system. Free 2',3'-cGAMP was generated in-house or obtained from APExBIO. ADU-S100 was obtained as a gift from Novartis. STF-1084 and endocytosis inhibitors were obtained from Sigma-Aldrich. No unexpected or unusually high safety hazards were encountered.

Equipment and Instrumentation. Protein purification was performed using a Cytiva AKTA go fast protein liquid chromatograph (FPLC). Colorimetric 96-well plate measurements were performed on an Agilent BioTek Synergy H1 plate reader. Small molecules were purified on a Biotage Isolera One flash chromatography system. Dynamic light scattering (DLS) was performed on a Horiba nanoPartica SZ-100V2. Flow cytometry measurements were performed on a ThermoFisher Attune NxT flow cytometer at the QB3 Cell and Tissue Analysis Facility (CTAF), UC Berkeley. RT-qPCR was performed using a BioRad CFX Connect thermocycler, also part of QB3 CTAF, UC Berkeley.

Liquid Chromatography/Mass Spectrometry (LC/MS). Purified proteins and small molecules were analyzed by electrospray ionization time-of-flight mass spectrometry (ESI-TOF-MS). Samples were first separated with an elution gradient of milli-Q H_2O + 0.1% (v/v) formic acid and Optima MS grade MeCN + 0.1% (v/v) formic acid, on an Agilent PLRP-S 1000 Å monolithic analytical column using an Agilent 1260 series liquid chromatography (LC) system, then mass spectra were obtained on an Agilent 6530 Q-TOF MS system.

Mammalian Cell Culture. THP-1 reporter cells were cultured in RPMI + GlutaMAX supplemented with 10% FBS. MC38 cells were cultured in DMEM + GlutaMAX supplemented with 10% FBS. All cells were maintained at 37 °C in a humidified environment and 5% CO_2.

***In vivo* Mouse Experiments.** All studies were performed according to protocols approved by the UC Berkeley Animal Care and Use Committee (ACUC). C57BL/6J and STING$^{gt/gt}$ mice were purchased from Jackson Laboratory and maintained at UC Berkeley.

Protein Preparation and Sequences:

Expression and Purification of MS2 Constructs. MS2 capsid sequences were expressed and purified based on a protocol adapted from a previous work[39,43]. Briefly, a single colony of DH10b *E. coli* containing a pBAD plasmid containing the gene for each MS2 variant was grown overnight in a 10 mL culture of LB at 37 °C, then subcultured into 1 L of 2xYT media with 20 µg/mL chloramphenicol and incubated shaking at 37 °C. OD_{600} was monitored until reaching a value of

bioRxiv preprint doi: https://doi.org/10.64898/2026.06.26.734859; this version posted July 1, 2026. The copyright holder for this preprint (which was not certified by peer review) is the author/funder, who has granted bioRxiv a license to display the preprint in perpetuity. It is made available under a CC-BY 4.0 International license.

0.6, upon which 0.1% (w/v) arabinose was added and cells grown overnight. The next day, cells were collected by centrifugation and resuspended in 20 mL of 10 mM sodium phosphate, pH 7.4 + 0.02% (w/v) NaN_3, and lysed on ice by sonication at 75% amplitude for 10 min (2 s on, 4 s off). The cell lysate was then centrifuged at 14,000 x g for 30 min and supernatant collected. MS2 protein was precipitated by addition of an equal volume of saturated aqueous $(NH_4)_2SO_4$ and rotation overnight at 4 °C. The mixture was then centrifuged at 14,000 x g for 30 min and supernatant was discarded. The precipitate was redissolved in 10 mM sodium phosphate, pH 7.4 + 0.02% NaN_3, centrifuged again to remove undissolved material, and filtered through a 0.22 µm membrane filter. Sample was then purified by FPLC first using two HiScreen CaptoCore 700 columns connected in series, with an isocratic flow of the same phosphate buffer. The flow-through was then desalted into 20 mM sodium phosphate buffer, pH 7.4, using a HiPrep 26/10 Desalting column. For PKR MS2, samples were further purified by FPLC on a 5 mL HiTrap Heparin HP affinity column, eluting with a gradient of 20 mM sodium phosphate, pH 7.4 (buffer A) to 20 mM sodium phosphate + 2 M NaCl, pH 7.4 (buffer B). The purified MS2 was then buffer exchanged into phosphate-buffered saline (PBS) using an Amicon 100kDa MWCO spin concentrator. Purified MS2 was confirmed by SDS-PAGE and LC/MS and stored at 4 °C.

Expression and Purification of SUMO-cGAS. A pET-29b(+) plasmid vector containing the gene for His6-SUMO-cGAS was purchased from Twist Biosciences and transformed into BL21(DE3)* *E. coli* cells. The transformed cells were plated on kanamycin-containing agar overnight, and a single colony was inoculated into 5 mL LB media and grown overnight at 37 °C. The sample was then subcultured into 1 L of TB media with 50 µg/mL kanamycin and grown at 37 °C until OD_{600} reached 0.6, at which point the sample was cooled to 16 °C. Once equilibrated at 16 °C, 0.1 mM IPTG was added and cells were grown for 16 h. Cells were then harvested by centrifugation and dissolved in freshly prepared lysis buffer (50 mM Tris, 300 mM NaCl, 30 mM imidazole, 1 mM TCEP, pH 8.0) with PMSF added to 1 mM. Cells were sonicated on ice at 75% amplitude for 10 min (2 s on, 4 s off), then centrifuged at 16,000 x g for 30 min. The supernatant was collected and filtered through a 0.22 µm PES syringe filter. An AKTA go FPLC was first cleaned by flushing all lines with 70% acetic acid in water, then 2 N NaOH, then milli-Q H_2O to remove all potential contaminants. The sample was then purified on a 5 mL HisTrap HP, using a gradient of lysis buffer (buffer A) and the same buffer with 300 mM imidazole (buffer B). The purified protein was then desalted into 50 mM Tris, 1 mM TCEP, pH 8.0 without added NaCl using a HiPrep 26/10 desalting column. Care was taken to minimize NaCl concentration from pH adjustment, as this was observed to inhibit cGAS activity. Purified SUMO-cGAS was confirmed by LC/MS. Protein was either used immediately or snap-frozen in 10% glycerol and stored at -80 °C.

bioRxiv preprint doi: https://doi.org/10.64898/2026.06.26.734859; this version posted July 1, 2026. The copyright holder for this preprint (which was not certified by peer review) is the author/funder, who has granted bioRxiv a license to display the preprint in perpetuity. It is made available under a CC-BY 4.0 International license.

Protein Sequences:

For MS2 sequences, engineered residues are underlined.

MS2 N87C – molecular weight: 13,717 Da

ASNFTQFVLVDNGGTGDVTVAPSNFANGVAEWISSNSRSQAYKVTCSVRQSSAQNRKYT
IKVEVPKVATQTVGGVELPVAAWRSYLCMELTIPIFATNSDCELIVKAMQGLLKDGNPIPS
AIAANSGIY

MS2 S37P T71K G73R N87C (PKR MS2) – molecular weight: 13,854 Da

ASNFTQFVLVDNGGTGDVTVAPSNFANGVAEWISSNPRSQAYKVTCSVRQSSAQNRKYT
IKVEVPKVATQKVRGVELPVAAWRSYLCMELTIPIFATNSDCELIVKAMQGLLKDGNPIPS
AIAANSGIY

SUMO-cGAS – molecular weight: 55,740 Da (truncated human cGAS sequence underlined)

GSSHHHHHHSSGLVPRGSHMSDSEVNQEAKPEVKPEVKPETHINLKVSDGSSEIFFKIKK
TTPLRRLMEAFAKRQGKEMDSLRFLYDGIRIQADQTPEDLDMEDNDIIEAHREQIGGMG
ASKLRAVLEKLKLSRDDISTAAGMVKGVVDHLLLRLKCDSAFRGVGLLNTGSYYEHVKI
SAPNEFDVMFKLEVPRIQLEEYSNTRAYYFVKFKRNPKENPLSQFLEGEILSASKMLSKF
RKIIKEEINDIKDTDVIMKRKRGGSPAVTLLISEKISVDITLALESKSSWPASTQEGLRIQN
WLSAKVRKQLRLKPFYLVPKHAKEGNGFQEETWRLSFSHIEKEILNNHGKSKTCCENKE
EKCCRKDCLKLMKYLLEQLKERFKDKKHLDKFSSYHVKTAFFHVCTQNPQDSQWDRK
DLGLCFDNCVTYFLQCLRTEKLENYFIPEFNLFSSNLIDKRSKEFLTKQIEYERNNEFPVFD
EF

Experimental Protocols:

Enzymatic Synthesis and Purification of 2',3'-cGAMP. In a 500 mL solution of 50 mM Tris, pH 8.0, without added NaCl was added 2 mM ATP, 2 mM GTP, 10 mM $MgCl_2$, 0.1 mg/mL herring testis DNA, 1 mM TCEP, and 1 μM cGAS. The sample was stirred for 24 h at 37 °C, after which the solution usually turned uniformly cloudy. The sample was then frozen and lyophilized and redissolved in 10 mL milli-Q water. The cloudy solution was centrifuged to remove precipitate, and the supernatant was collected, filtered through a 0.22 μm PES syringe filter, then purified on a 15.5 g ISCO C18aq flash chromatography cartridge, using a gradient of 50 mM triethylammonium acetate (TEAA) in water and MeCN. Purified cGAMP eluted at approximately

bioRxiv preprint doi: https://doi.org/10.64898/2026.06.26.734859; this version posted July 1, 2026. The copyright holder for this preprint (which was not certified by peer review) is the author/funder, who has granted bioRxiv a license to display the preprint in perpetuity. It is made available under a CC-BY 4.0 International license.

15% MeCN, and fractions were collected, verified using LC/MS, and lyophilized to obtain pure cGAMP.

Construction of MS2-cGAMP. MS2 N87C was diluted in DPBS to 50 µM, and 3 equiv. cGAMP-disulfide was added. The sample was incubated at 4 °C for at least 1 h, after which complete conjugation was confirmed by LC/MS. Excess cGAMP was then removed by buffer exchanging into clean DPBS sequentially 5 times using a 0.5 mL Amicon 100 kDa MWCO spin concentrator. The resulting samples were then filtered through a 0.22 µm cellulose acetate centrifuge filter.

THP-1 STING reporter cell experiments. THP-1 cells bearing a tdTomato STING reporter and, where applicable, a SLC19A1 knockout were generated in a previous study[19]. For STING KD reporter cells, THP-1 monocytes were transduced with 1) a lentiviral dCas9-HA-BFP-KRAB-NLS expression vector (Addgene, plasmid no.102244), 2) a lentiviral vector (pCRISPRia-v2, Addgene, plasmid no. 84832) expressing a control gRNA (GGAGAGACGGTACCGTCTCA) or STING-targeting gRNA (GGCTGCTCTGGATGATGACG) and 3) a lentiviral vector encoding the tdTomato reporter gene driven by the ISREs and the minimal mouse IFN-β promoter.

For STING agonist treatments, THP-1 STING reporter cells were spun down, resuspended in fresh media with or without FBS or inhibitor, and plated in a 48-well plate at 50,000 cells/well and treated with a 10x dilution of MS2-cGAMP, free cGAMP, or free ADU-S100 in DPBS for 24 h. Cells were then transferred to a V-bottom 96-well plate and analyzed by flow cytometry. Cells were gated first by FSC/SSC, then doublets removed by FSC-A/FSC-H gating, followed by presence of a constitutive GFP signal in cells with the STING reporter, followed by tdTomato analysis.

THP-1 STING reporter cell uptake of fluorescently labeled MS2. AZdye 647-maleimide was obtained from Vector Labs and dissolved to 10 mM in DMSO. PKR MS2 was incubated with 3 equiv of AZdye 647-maleimide at pH 7.2 for 1 h at 4 °C, then analyzed by LC/MS to confirm complete modification. Samples were then desalted using a 0.5 mL Zeba 40 kDa spin desalting column into DPBS. THP-1 cells were then plated at 50,000 cells/well in a 48-well plate and treated with 0.2 µM MS2-dye for 24 h. Cells were then transferred to a V-bottom 96-well plate and analyzed by flow cytometry using identical gating as before and analysis of the AlexaFluor 647 channel.

THP-1 STING reporter endocytosis inhibitor screen. THP-1 cells were plated in a 48-well plate at 50,000 cells/well and pre-incubated with 10 U/mL heparin, 1 U/mL heparinase, 50 µM chloroquine, 5 µM cytochalasin D, or 80 µM dynasore for 2 h. Cells treated with heparinase were spun down, media was removed by aspiration, and fresh media was replaced. Cells were then treated with fluorescently labeled MS2 for uptake or STING agonists for STING activation and analyzed as described above.

RT-qPCR of STING agonist-treated cells. RT-qPCR conditions were adopted from a previous study[40]. Briefly, THP-1 or MC38 cells were treated with 100 nM PKR-cGAMP, 100 µM free

bioRxiv preprint doi: https://doi.org/10.64898/2026.06.26.734859; this version posted July 1, 2026. The copyright holder for this preprint (which was not certified by peer review) is the author/funder, who has granted bioRxiv a license to display the preprint in perpetuity. It is made available under a CC-BY 4.0 International license.

cGAMP, or 10 μM free ADU-S100 for 4 or 24 h. Adherent MC38 cells were then lifted by 0.25% trypsin. All cell types were then centrifuged, and media was removed by aspiration. RNA was extracted using TriZOL buffer and chloroform per manufacturer instructions, and the resulting RNA was converted to cDNA using a BioRad iScript cDNA synthesis kit. A 350 ng sample of cDNA was mixed with SYBR Green Master Mix and 500 nM of primer in 20 μL nuclease-free water. qPCR data was obtained using the following conditions on a thermocycler: 95 °C for 2 min, followed by 42 repeats of 95 °C for 15 s, followed by 60 °C for 60 s. Primer sequences: Mouse *IFNB* fwd: 5'-ATAAGCAGCTCCAGCTCCAA-3', rev: 5'-CTGTCTGCTGGTGGAGTTCA-3'; Human *IFNB* fwd: 5'-AAACTCATGAGCAGTCTGCA-3', rev: 5'-AGGAGATCTTCAGTTTCGGAGG-3'; *ACTB* (endogenous housekeeping control) fwd: 5'-AGAGCTACGAGCTGCCTGAC-3', rev: 5'-AGCACTGTGTTGGCGTACAG-3'.

Cell viability assay. THP-1 cells were treated with STING agonists or controls as described above. After 24 h, samples were split in half, with half the samples analyzed by flow cytometry to ensure expected STING activation. The other half of each well was transferred to a 96-well U-bottom plate, where cells were centrifuged and media removed by aspiration. MTS reagent was obtained from APExBIO (K2250) and diluted 10x in DPBS. 200 μL diluted reagent was added to each well and cells were incubated and analyzed according to the manufacturer's instructions.

PMA differentiation of THP-1 STING reporter cells. THP-1 cells were differentiated based on a previous study[40]. Cells were mixed with 125 ng/mL of phorobol 12-myristate 13-acetate and plated in a 48-well plate at 50,000 cells/well. Cells were incubated for 48 h, after which differentiated cells had adhered to the plate bottom. Media was aspirated and replaced with PMA-free RPMI media, in which cells were incubated for 24 h. Cells were then treated with STING agonists and controls as previously. Cells were lifted with 40 μL 0.25% trypsin and diluted with RPMI, after which they were analyzed by flow cytometry.

***In vivo* antitumor studies.** For *in vivo* tumor inoculation, cells were washed and resuspended in PBS, and 50 μL containing 4×10^6 cells was injected subcutaneously into C57BL/6J and $STING^{gt/gt}$ mice. Tumor dimensions (length, width, and height) were measured using digital calipers, and tumor volume was calculated using the ellipsoid formula: $V = (\pi/6) \times \text{length} \times \text{width} \times \text{height}$. When tumors reached a volume of 50 mm^3, they were injected intratumorally with PBS, PKR MS2-cGAMP corresponding to 0.2, 1, 2, or 5 μg cGAMP, 50 μg ADU-S100, or 50 μg free cGAMP in 50 μL PBS. In all experiments, prior to treatment initiation, tumor-bearing mice were stratified by tumor volume and randomly assigned to treatment groups such that mean tumor sizes were comparable across groups. Mice were euthanized upon reaching institutional humane endpoints.

bioRxiv preprint doi: https://doi.org/10.64898/2026.06.26.734859; this version posted July 1, 2026. The copyright holder for this preprint (which was not certified by peer review) is the author/funder, who has granted bioRxiv a license to display the preprint in perpetuity. It is made available under a CC-BY 4.0 International license.

Synthetic Methods:

Synthesis of cGAMP-disulfide:

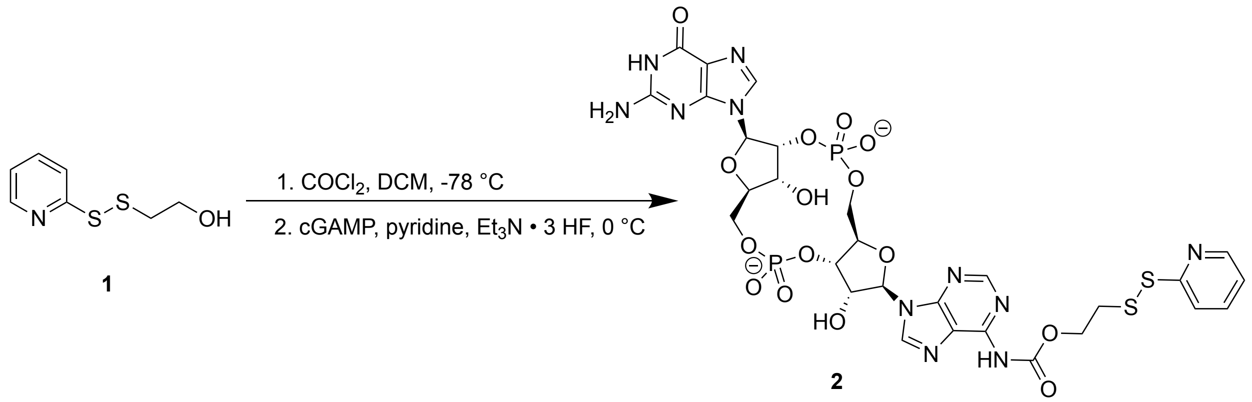

Scheme S1. Synthesis of cGAMP-disulfide conjugate.

cGAMP-disulfide conjugate 2

Synthesis of the pyridyl disulfide compound **1** was performed as in a previous study[40]. Compound **1** (42 mg, 0.223 mmol) was dissolved in 1 mL of anhydrous DCM. A solution of phosgene (15% in toluene, 0.54 mL, 0.742 mmol) was added dropwise and the reaction was stirred at 0 °C under N_2 for 1 h. Solvent and excess phosgene were then removed by vacuum. About 1 mL of dry DCM was added and then removed under vacuum 3 times to azeotropically remove any excess phosgene. A portion of 2',3'-cGAMP (5 mg, 0.00742 mmol) was suspended in 1 mL of dry pyridine, and triethylammonium trihydrofluoride (Et_3N • 3 HF) was added in 25 µL increments until the solids were fully dissolved, with roughly 100-150 µL HF-TEA needed. The solution was then added to the reaction mixture, and the resulting reaction was stirred for 90 min at 0 °C under N_2. The volatile components were then evaporated under vacuum, and the reaction was quenched with 500 µL water, then dissolved in 1.5 mL DMSO. The product was purified twice by Biotage C18 column chromatography (50 mM NEt_3•AcOH in H_2O/MeCN) with elution at 30% MeCN. Concentration of the fractions yielded **2** as a white solid (0.7 mg, 11% yield).
